# Novel sensing technology in fall risk assessment in older adults: a systematic review

**DOI:** 10.1186/s12877-018-0706-6

**Published:** 2018-01-16

**Authors:** Ruopeng Sun, Jacob J. Sosnoff

**Affiliations:** Department of Kinesiology and Community Health, University of Illinois at Urbana-Champaign, 301 Freer Hall, 906 S Goodwin Ave, Urbana, 61801 USA

**Keywords:** Geriatric, Older adults, Fall risk, Sensing technology

## Abstract

**Background:**

Falls are a major health problem for older adults with significant physical and psychological consequences. A first step of successful fall prevention is to identify those at risk of falling. Recent advancement in sensing technology offers the possibility of objective, low-cost and easy-to-implement fall risk assessment. The objective of this systematic review is to assess the current state of sensing technology on providing objective fall risk assessment in older adults.

**Methods:**

A systematic review was conducted in accordance to the Preferred Reporting Items for Systematic Reviews and Meta-Analysis statement (PRISMA).

**Results:**

Twenty-two studies out of 855 articles were systematically identified and included in this review. Pertinent methodological features (sensing technique, assessment activities, outcome variables, and fall discrimination/prediction models) were extracted from each article. Four major sensing technologies (inertial sensors, video/depth camera, pressure sensing platform and laser sensing) were reported to provide accurate fall risk diagnostic in older adults. Steady state walking, static/dynamic balance, and functional mobility were used as the assessment activity. A diverse range of diagnostic accuracy across studies (47.9% - 100%) were reported, due to variation in measured kinematic/kinetic parameters and modelling techniques.

**Conclusions:**

A wide range of sensor technologies have been utilized in fall risk assessment in older adults. Overall, these devices have the potential to provide an accurate, inexpensive, and easy-to-implement fall risk assessment. However, the variation in measured parameters, assessment tools, sensor sites, movement tasks, and modelling techniques, precludes a firm conclusion on their ability to predict future falls. Future work is needed to determine a clinical meaningful and easy to interpret fall risk diagnosis utilizing sensing technology. Additionally, the gap between functional evaluation and user experience to technology should be addressed.

## Background

Falls are the leading cause of accidental death and injury in older adults [1]. One in 3 older adults over the age of 65 and 1 in 2 over 85 years of age will experience a fall in the next year and a significant portion of those that fall will suffer an injury [2]. Given the adverse consequence of falls in older adults, considerable research has focused on identifying individual fall risk factors and targeted fall prevention [2–6]. This collective research has revealed that falls and fall-related injuries are predictable and preventable with interventions targeting modifiable risk factors such as muscle strength, balance and mobility [7]. It is also maintained that effective fall prevention programs are cost effective and an appropriate method to maximize quality of life and maintain independence of older adults [7]. The first step to an effective fall prevention program is to identify those at risk of falling and then to determine the most appropriate interventions to reduce or eliminate falls [2].

While the American Geriatric society as well as the Centers for Disease Control and Prevention (CDC) recommends screening of fall risk for older adults at least annually by physicians [8], effective fall risk screening is still underutilized and not routinely integrated into clinical practice. There are several reasons for the lack of fall risk assessment in current practice ranging from overreliance on unreliable subjective measures, lack of cost-effective assessment technology and clinical time constraints.

Therefore, accurate, inexpensive, easy to administer fall risk assessments that can be undertaken regularly are warranted. Novel technology, such as inertial sensors, smartphone, low-cost video/depth camera, pressure sensors and motion ambient sensors, offer an alternative approach that can efficiently capture and analyze movement data and may provide an easy-to-implement objective fall risk assessment. Inertial sensor usually contains miniaturized accelerometers and/or gyroscopes that quantify movement pattern/abnormality by various time and frequency domain parameters. Low cost video/depth sensing camera (i.e. Microsoft Kinect™) provides marker-less 3D motion tracking of body joints by using its built-in and externally validated human skeleton modelling algorithms. It successfully eliminates the need for markers and calibration procedures characteristic of traditional motion capture, thereby enabling fast and patient-friendly 3D body motion analysis [9]. Low cost pressure sensing platform (Wii board, pressure sensitive insole / pad, etc.) provides critical information on postural stability as well as temporal pattern of stepping/gait [10, 11]. Motion ambient sensing (Radar/Laser, etc.) technology can unobtrusively track movement of different body segments, and identify the movement abnormality in impaired individuals. Mobile phone has also been proposed as a potential instrument for balance/mobility tracking by using its built-in inertial sensor and/or camera [12–14].

To date, fall risk assessment technology has incorporated various screening tools, assessment activities, outcome variables, and fall discrimination/prediction models. There have been a few attempts to synthesize fall risk assessment using wearable sensing technology. A systematic review in 2013 by Howcroft and colleagues [15] focused entirely on inertial sensors use in fall risk assessment. It provided an overview of study methodologies, parameters derived and model effectiveness in faller prediction/discrimination, and concluded inertial sensors have the potential to provide objective fall risk assessment in older adults. Another review by Shany and colleagues in 2012 [16] provided a practical discussion on various issues concerning the use of using wearable sensors in a fall risk assessment context (i.e. whether it should or can be used in unsupervised environment). In a separated review, Shany et al. [17] raised concerns of over-inflated diagnostic accuracy in wearable sensor-based fall risk testing, primarily due to small sample size, questionable modelling decisions and inappropriate validation methodologies. However, there is a lack of knowledge about the use of other technologies (non-wearable) in fall risk assessment. Rapid technology advancements and additional research in recent years necessitates a timely systematic review of technology used in fall risk assessment. Consequently, the aim of this paper is to systematically evaluate the use of technology in performing fall risk assessments, and more specifically, to evaluate the test, sensor, algorithm effectiveness on predicting and/or discriminating older adult fallers from non-fallers.

## Methods

### Search strategy and criteria

This systematic review was conducted in accordance to the Preferred Reporting Items for Systematic Reviews and Meta-Analysis statement (PRISMA) [18]. Keyword search was performed in PubMed, Web of Science, Cochrane Library and CINAHL on May 2017. The search algorithm included all possible combinations of keywords (with wildcard characters and MeSH term) from the following 4 groups: (fall risk* OR fall predict* OR fall screen* OR fall assess*) and (walk OR locomot* OR ambulat* OR mobility OR gait OR balance OR postural OR posture OR reaction time OR strength) and (Aged OR geriatr* OR gerontol* OR senior OR elder* OR old*) and (acceler* OR inertia* OR gyro* OR wearable OR camera OR sens* OR phone* OR technolog* OR tool OR instrument*), Whereas articles with one or more of the following keywords were excluded: (exercise OR Intervention OR training OR free living OR daily OR activity). Reference lists from the identified publications were reviewed to identify additional research articles of interest.

Studies that met all of the following criteria were included in the review: 1) peer-reviewed publication in English language; 2) published since 2011 (to avoid redundant overlapping results with previous reviews conducted in 2012 [16] and 2013 [15]); 3) objective fall risk assessment conducted using commercially available technology (Inertial sensor, Video/depth Camera, Wii balance board, smartphone, pressure sensing insole, ambient sensing); 4) involvement of a geriatric population (based on a mean participant age greater than 60 years); 5) fall risk identified through accepted methods (physician screening, fall history, prospective fall incident tracking, or proven clinical fall risk assessment tool (Berg Balance Scale - BBS, Timed Up and Go - TUG, Short Physical Performance Battery - SPPB, Physiological Profile Assessment - PPA, Tinetti Performance Oriented Mobility Assessment - Tinetti, etc)); 6) the fall risk assessment involves a structured routine of movement sequence, and not free daily-living activity tracking; 7) evaluate the effectiveness (i.e. accuracy, specificity, sensitivity) [19] of the technology in identifying high risk faller. Studies were excluded from the review if they meet one or more of the following criteria: 1) Proof of concept on the technology use in movement tracking; 2) technologies that focused on fall detection; 3) studies that investigated fall risk solely in a neurological impaired population.

**Fig. 1:**
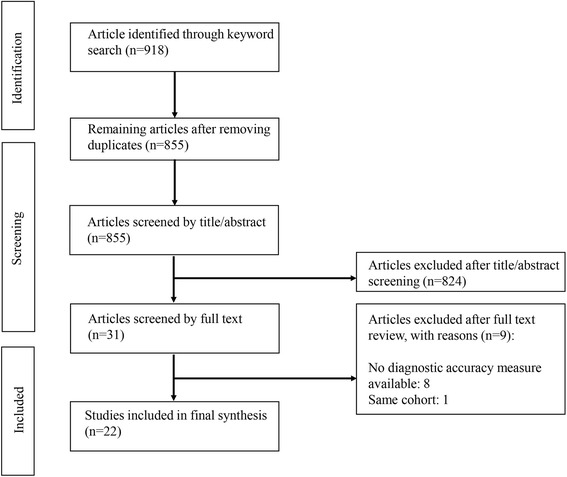
Article selection flow chart

Titles and abstracts of the articles identified through keyword search were screened against the study selection criteria. Potentially relevant articles were retrieved for evaluation of the full text. The results of this search are shown in Fig. 1.

## Results

### Study selection

Figure 1 depicts the article identification and selection process. Eight hundred fifty-five unduplicated articles were identified through keyword and reference search. Eight hundred twenty-four articles were excluded after title and abstract screening. The remaining 31 articles were full text read. A total of 9 articles were excluded due to following reasons: 1) lack of diagnostic accuracy measures (*n* = 8), 2) duplicated cohort (i.e. studies based on identical samples) in assessment (*n* = 1). The remaining 22 articles were included in the review [10, 20–33].

### Study analysis

Table 1 list the pertinent methodological features (faller identification method, participant demographics, sensor technology employed, sensor placement location if applicable, structured movement sequence used for fall risk assessment, outcome parameters used, modelling technique and performance for faller discrimination/prediction) from each included article.

**Table 1:** Study Characteristics

Author/Year	Faller Identification Method	Population/Sample Size/Age (Mean ± SD)	Technology	Sensor placement if applicable	Test Protocol	Outcome Measures	Model	Model validation	Accuracy	Specificity	Sensitivity	AUC
Bautmans et al. 2011 [34]	Fall history (> = 1) in past 6- month, or TUG >15 s, or Tinetti <=24	F (n = 40, 80.6 ± 5.4), NF (*n* = 41, 79.1 ± 4.9), YA (*n* = 40, 21.6 ± 1.9)	A single Tri-axial Accelerometer	Sacrum	Straight line walking	Gait Speed, Step time symmetry, step/stride regularity	Logistic regression. Only gait speed was effective for discrimination analysis	NA	77	78	78	0.83
Caby et al. 2011 [35]	Fall history (> = 1) in past 1-year, with additional physician screening	F (*n* = 15, 80.1 ± 5.3), NF (n = 5, 83.2 ± 4.3)	10 Tri-axial Accelerometer sensor network	Knee, Ankle, Elbow, Wrist, Shoulder	Straight line walking	67 gait acceleration features extracted(temporal, frequency, power, and correlation between sensors)	RBFN SVM, KNN, NB	Leave-one-out cross validation	75-100	40-100	93-100	
Jansen et al. 2011 [36]	Fall history (> = 1) unknown length, or TUG >15 s, or Tinetti <=24	F (n = 40, 80.6 ± 5.4), NF (n = 40, 79.0 ± 5.0)	A single Tri-axial Accelerometer	Sacrum	Straight line walking	22 acceleration features 5 groups (step count, step time, step length, step symmetry and step RMS)	NB,MLP,SVM, LWL, Decision Tree, NEAT	Ten-fold cross validation (Max value)	61-82	62-84	58-80	
Liu et al. 2011 [37]	Fall history (> = 1) in past 1-year	OA (*n* = 68, 80.1 ± 4.4; MF/NMF = 9/59) MF (> = 2 falls)	A Tri-axial Accelerometer	Waist	TUG,AST,STS5	126 features (temporal, energy, spectral)	Linear multiple regression,	Leave-one-out cross validation	78	90	59	
Marschollek et al. 2011 [38]	1-year prospective fall occurrence (> = 1)	OA (*n* = 46, 81.3; F/NF = 19/27)	A Tri-axial Accelerometer	Waist	TUG, Straight line walking	Kinetic Energy, Pelvis Sway, Gait variability, Step time/length, number of steps for TUG, spectral density parameters	Decision tree, logistic regression	Ten-fold cross validation (mean value)	65-80	78-96	42-74	0.65-0.87
Paterson et al. 2011 [40]	1-year prospective fall occurrence (> = 1)	F (*n* = 54, 69.0 ± 6.9) NF (*n* = 43, 68.4 ± 7.3)	Two Tri-axial Accelerometers	Foot mount	7 min walking on a circuit	Stride dynamic (Fractal Scaling Index)	Logistic regression	NA	67	58.1	74.1	
Weiss et al. 2011 [39]	Fall history, past 1-year (> = 2)	F (*n* = 23, 76.0 ± 3.9) NF (*n* = 18, 68.3 ± 9.1)	A Tri-axial Accelerometer	Lower back	TUG	Duration of TUG and subtasks, acceleration range and Jerk. Number of steps for TUG, gait speed	Logistic regression	NA	63.4-87.8	50.0-83.3	65.2-91.3	
Yamada et al. 2011 [20]	Fall history (> = 1) in past 1-year	F (*n* = 16, 84.8 ± 10.1) NF (*n* = 29, 80.2 ± 6.4)	Wii Balance Board	NA	Game-based measure in seated/standing	Game score	Discriminate analysis	NA	88.6			
Greene et al. 2012 [21]	2-year prospective fall occurrence (> = 2)	F (*n* = 83, 71.8 ± 6.9) NF (*n* = 143, 71.4 ± 6.6)	Two Tri-axial Inertial sensors (accelerometer/gyroscope)	Shank	TUG	44 features (spatial/temporal gait parameters, angular velocity parameters, turn parameters)	Discriminate classifier	Ten-fold cross validation (mean value)	73-83	73-96	56-90	0.74-0.85
Greene et al. 2012 [22]	Fall history (> = 2, or one fall requiring medical attention) in past 1-year	F (*n* = 65, 74.0 ± 5.8) NF (*n* = 55, 73.3 ± 5.8)	A Tri-axial Inertial sensor (accelerometer/gyroscope)	Lower back L3	Standing balance (EO/semi- tandem, EC/narrow stance)	RMS of AP/ML acceleration, frequency variability, spectral entropy	SVM	Ten-fold cross validation (mean value)	63-72	58-82	59-67	
Schwesig et al. 2012 [23]	1 year prospective fall occurrence (> = 1)	OA (*n* = 141, 82.7; MF/NMF = 17/124, MF (> = 3 falls)	Two Tri-axial Inertial sensors (accelerometer/gyroscope)	Shoe-mounted	Straight line walking	Temporal gait parameters	Logistic regression, ROC curve	NA		42-61	63-100	0.66-0.7
Senden et al. 2012 [24]	Tinetti <=24	F (*n* = 50, 79 ± 6) NF (*n* = 50, 74 ± 5)	A Tri-axial Accelerometer	Sacrum	Straight line walking	spatial-temporal gait parameters, step time symmetry, harmonic ratio, inter-stride variability, RMS acceleration	Linear regression, ROC curve	NA				0.67-0.85
Doheny et al. 2013 [25]	Fall history, past 1-year (> = 2, or one fall requiring medical attention)	F (*n* = 19, 74.9 ± 7.0) NF (*n* = 20, 68.4 ± 6.2)	Two Tri-axial Inertial sensors (accelerometer/gyroscope)	Sternum, Thigh	STS5	Total Time, Sub-phase time, Spectral Edge Frequency, postural sway (RMS acceleration),	Logistic regression	Leave-one-out cross validation	74.4	80	68.7	0.70
Doi et al. 2013 [26]	1 year prospective fall occurrence (> = 1)	F (n = 16, 84.8 ± 5.9) NF (*n* = 57, 79.7 ± 8.2)	Two Tri-axial Accelerometer	Upper/lower trunk	Straight line walking	Harmonic Ratio	Logistic regression, ROC curve	NA		84.2	68.8	0.81
Riva et al. 2013 [28]	Fall history (> = 1) in past 1-year	F (*n* = 44, 63.3 ± 6.4) NF (*n* = 90, 62.0 ± 6.1)	Tri-axial Accelerometer	Lower back	Treadmill walking	Harmonic Ratio, Index of harmonicity, Multiscale Entropy, Recurrence quantification analysis parameters	Logistic regression	NA	71-72.5	96.6	16.7-21.4	
Nishiguchi et al. 2013 [27]	Fall history (> = 1) in past 1-year	F (n = 41, 75.4 ± 4.6) NF (*n* = 111, 73.5 ± 4.6)	Laser Range Finder	NA	Choice Stepping Test	Step reaction time, error rate, stepping –response score	Logistic regression, ROC curve	NA		69.7	73.0	0.73
Colagiorgio et al. 2014 [29]	Combination of (Tinetti + BBS + BESTest) < 29 / 33	OA (*n* = 66, 76 ± 10, F/NF = 22/44) YA (*n* = 13, 26 ± 5)	Microsoft Kinect	NA	Standing balance(EO,EC, Nudged on firm surface or foam surface), Reaching forward, Stand-to -Sit, Sit-to- Stand, AST	80 features (COM postural sway, Chest Pitch Angle, velocity of transition, velocity of stepping)	Majority Classifier, Decision Tree, SVM,KNN, NB	.632 bootstrap technique	47.9-84.3	47.8-91.3	47.7-83.1	
Simila et al. 2014 [31]	BBS < =49	OA (n = 20, 76.8 ± 5.6) YA (n = 19, 27.5 ± 4.4) NP (n = 15, 55.2 ± 7.3)	Tri-axial Accelerometer	Lower back	BBS, straight line walking	Resultant acceleration in each task, gait pattern as measured by averaged acceleration in each step	KNN, ROC curve	NA	60.8-87.2	62-96.6	42.1-89.5	0.66-0.89
Kargar et al. 2014 [30]	Physician examination	OA (*n* = 12, 65 -90; F/NF = 7/5)	Microsoft Kinect	NA	TUG	Number of steps of TUG, step time, turn duration	SVM	Leave-one-out cross validation	67.4	67.5	67.3	
Kwok et al. 2015 [32]	1 year prospective fall occurrence (> = 1)	F (n = 18, 70.7 ± 5.2) NF (n = 55, 69.7 ± 7.8)	Wii balance board	NA	Standing balance (EO)	Mean sway velocity	Logistic regression, ROC curve	NA				0.67-0.71
Howcroft et al. 2016 [10]	Fall history (> = 1) in past 6-month	F (*n* = 24, 76.3 ± 7.0) NF (*n* = 76, 75.2 ± 6.6)	Pressure sensing insole, Tri-axial Accelerometers	Head, Pelvis, Shank, Shoe	Single/Dual task straight line walking	COP path parameters, temporal gait parameters, Harmonic Ratio, Maximum Lyapunov exponent(local dynamic stability)	MLP, NB, SVM	Hold out method (75% training set, 25% test set)	72-84	73.7-100	33.3-100	
Howcroft et al. 2017 [33]	6- month prospective fall occurrence (> = 1)	F (*n* = 28, 75.0 ± 8.2) NF (*n* = 47, 75.3 ± 5.5)	Pressure sensing insole, Tri-axial Accelerometers	Head, Pelvis, Shank, Shoe	Single/Dual task straight line walking	COP path parameters, temporal gait parameters, Harmonic Ratio, Maximum Lyapunov exponent(local dynamic stability)	MLP, NB, SVM	Hold out method (75% training set, 25% test set)	49.2-56.5	52.7-66.6	27.0-46.3	

*OA* Older Adult, *YA* Young Adult, *NP* Neurological Patient, *F* Faller, *NF* Non-Faller, *MF* Multiple Fa*ller; NMF Non-Multiple Faller*

### Faller identification

Individuals at high risk of falls were identified with various techniques including retrospective fall history (11/22), prospective fall occurrence (7/22), validated clinical assessment (5/22), or physician exam (1/22). Two studies combined the retrospective fall history and clinical assessments scores to identify high-risk fallers [34, 35]. The length of fall history recall (6 months to 1 year) and prospective fall occurrence follow up period (6 months to 2 years), as well as the number of falls chosen for faller classification (at least one fall, more than 2 falls, one fall requiring medical attention) also varied between studies.

### Sensor/activities used for fall risk assessment

Inertial sensors were used in 17 investigations. Of those 17, 11 utilized a sensor placed on the lower back area for quantifying center of mass movement [10, 22, 24, 26, 31, 33, 34, 36–39]. Six studies utilized sensors placed bilaterally on lower limb (foot/shoe/shank/ankle) for recording spatial/temporal gait parameters [10, 21, 23, 33, 35, 40], and 3 used a body sensor network (>3 sensors on various anatomical landmarks: head, shoulder, wrist, elbow, knee, pelvis, ankle/shank) for quantifying limb and trunk kinematics [10, 33, 35]. Sternum sensor has also been used for quantifying trunk movement [25, 26], while thigh sensor was used in one study for quantifying the transition of sitting to standing [25]. In all studies, inertial sensors were secured on participant via belt, tape or band.

Given inertial sensor’s unique benefit in unconstrained range of measurement, steady state walking (over ground/treadmill) was used as the primary assessment activity for 12 studies [10, 23, 24, 26, 28, 31, 33–36, 38, 40], while the Timed Up and Go (TUG) (*n* = 4) [21, 37–39], five time sit to stand(5STS) (*n* = 2) [25, 37], standing balance (*n* = 1) [22] and alternate step test (AST) (n = 1) [37] were also used in the assessment procedure.

Two depth camera (e.g. Kinect™) based fall risk assessment studies were identified [29, 30]. Due to its capture range (0.5 m −4.5 m), TUG [30], Standing balance, Sit-to-Stand transition and AST [29] were used as the fall risk assessment activities.

Two force-sensitive platform (Wii balance board) based fall risk assessment studies were identified [20, 32]. The tasks involved were standing balance [32] and seated/standing Wii balance gaming [20]. One laser-based fall risk assessment study used the choice stepping test as the fall risk screening activity [27].

Two studies done by the same research group utilized a pressure-sensing insole in conjunction with the inertial body-sensor network for steady-state walking assessment [10, 33].

### Outcome variables extracted

For steady state walking assessment, 8 of 12 studies reported spatial-temporal gait parameters (step time, step length, etc) and/or its variability (SD, CV) [10, 23, 24, 33, 35, 36, 38, 40], 5 of 11 studies reported harmonic ratio (an indicator for gait smoothness and overall stability) [10, 24, 26, 28, 33]. Other variables include gait speed [34], energy-related measures [38], frequency-domain measures [28, 38] local dynamic stability [10, 33], stride fractal scaling index [40] and gait symmetry [10, 24, 33, 34].

For test involving TUG assessment, extracted variables included time to complete the task and its subtasks (Sit-to-Stand, Stand-to-Sit, and turning) [37, 39], number of steps taken [30, 37, 39], cadence [37, 39],gait speed [37], temporal and spatial gait parameters and/or its variability [21, 30, 37, 39], acceleration/angular velocity/jerk amplitude [21, 37, 39], energy-related measures [37], and frequency-domain measures [37].

For standing postural sway assessment, the inertial sensor based study reported RMS acceleration, frequency variability, and spectral entropy as the outcome variables [22], whereas the depth camera based study reported COM sway area, sway velocity, and the stochastic parameter of COM sway [29]. The Wii based study reported COP sway velocity as the outcome measures [32].

For fall risk assessment involving other dynamic tests (alternate step test, forward reaching, Stand-to-Sit, Sit-to-Stand transition, dynamic balance game, and choice reaction stepping), outcome measures included the duration of movement [25, 27, 37],velocity of movement [29], acceleration amplitude [25, 31], jerk amplitude [37], energy-related measures [37], and frequency-domain measures [25, 37], reaction time [27], error rate [27] and specific performance scores [20, 27].

### Modelling technique and effectiveness in faller discrimination

A diverse collection of quantitative models/methods were employed to predict fall risk, which included: logistic regression, linear regression, Radial Basis Function Network classifier (RBFN), Support Vector Machine (SVM), Naïve Bayesian classifier, Multi-layer perceptron, Locally Weighted Learning, Decision Tree, Cluster analysis, k-Nearest Neighbor (kNN), neural networks, neuro evolution of augmenting topologies (NEAT), and discriminate analysis. Logistic regression was the most used modeling techniques (*N* = 10) for fall prediction modelling, although non-linear classification model was employed in recent publications.

Table 1 reports the effectiveness of diagnostic analysis (i.e. accuracy, sensitivity, specificity and area under curve for the ROC curve). Non-linear statistical classifier was primarily used in studies using inertial sensor and depth camera, due to the vast set of parameters extracted from the measured movement sequence. Whereas the Wii balance board and laser range finder measurement only provide a few features that require no advance modelling for fall risk identification. Due to the variation in assessment tools, movement routine, outcome variables extracted and modelling methods used, a diverse range of diagnostic performance was observed (Accuracy: 47.9-100%, Sensitivity: 16.7-100%, Specificity: 40-100%, AUC 0.65-0.89). In term of classification technique, naive Bayesian classifier, SVM, discriminate classifier, kNN, Decision tree, NEAT and logistic regression have been reported to achieve above 80% accuracy, although the direct comparison between classifiers cannot be achieved due to different tasks, parameters and populations. It is also worth noting that only 50% of studies were conducted with recommended model validation procedures such as Leave-one-out cross validation, Ten-fold cross validation, .632 bootstrap technique, and hold-out validation. Consequently, the diagnostic performance of studies without appropriate model validation could be over-inflated.

## Discussion

This systematic review examined the existing evidence from 2011 to 2017 regarding sensor technology used in fall risk assessment in older adults. By measuring movement during selected structured movements (walking, stepping, sit-to-stand, stand-to-sit, TUG, BBS, etc.), the sensing technology has been shown as viable assessment tool for fall risk assessment. Overall, these devices provide an accurate, inexpensive, and easy-to-administer objective fall risk assessment.

Over half of the reviewed papers used the retrospective fall history and/or clinical assessment tools as the standard for identifying individuals at high risk for falls. However, even though widely used as standard methods, the clinical assessment tools (TUG, Tinetti, BBS, etc.) still do not achieve 100% diagnostic accuracy. Furthermore, the retrospective fall recall may also suffer from the lack of reliability due to patient’s poor recollection [41]. In addition, a history of falling may lead to gait pattern changes due to injury or fear of falling [17]. Thus, prospective fall occurrence tracking should be utilized in future fall risk assessment research with a follow-up period of at least 6 months after initial assessment.

A vast number of movement-derived variables were reported in the inertial sensor and depth camera based fall risk assessment investigations. They ranged from duration of movement to movement smoothness. Such diverse measurements resulted in a diverse range of prediction/discrimination accuracy across studies. A large pool of variables may not be clinical relevant, or confounding with other existing variables [17], thus a selection of proper variables based on research evidence is necessary. Additionally, lack of appropriate model validation procedures (50% studies without cross-validation or holdout procedure) may yield over-inflated diagnostic accuracy, and are unlikely to maintain its reported performance during everyday use in relevant populations [17]. This observation highlights the need for proper guideline and standardized procedures of model construction/validation in future research [17].

It is worth noting that most if not all inertial sensor based fall risk assessment reviewed in this work used the sensor as a stand-alone recording device, thus require additional personnel to guide the user through the assessment routine, operate the system and interpret the data. Although with technological advances including increasing computing power, and integrated human interface devices (display, touch screen, voice command, etc.), this limitation may be minimized in the future. In contrast, the Kinect and Wii based systems, due to its compatible computer/gaming console, can provide automated interaction with the user, thus potentially allowing the user to complete the structured fall risk assessment without additional supervision. However, this unsupervised movement may raise other concerns such as safety and compliance.

Smartphone technology, although equipped with inertial sensors/camera, computing power and display to conduct an interactive assessment, has not been validated as a diagnostic tool for fall prediction [14]. However, numerous studies have validated its accuracy in balance and mobility tracking [12, 13], and several proof-of-concept have been proposed to investigate fall risk/ provide fall prevention intervention [42, 43].Other proof-of-concept studies that utilizing depth camera and radar sensing to provide in-home movement monitoring and fall risk assessment have also been reported in recent years [6, 44–46]. In the long term, such ambient sensing technology may provide an unsupervised, automated fall risk screening tool in a community and/or assisted living settings. Several studies have also reported that IMU sensor and wireless pressure insole devices can continuously monitor daily-living activity, and derive gait parameters for diagnostic proposes [11, 47].

The primary goal of all existing technologies is to facilitate the identification of those at a risk of falling, and thus provide appropriate fall prevention intervention. Only limited investigation has been conducted to understand the seniors’ acceptance in using the technology [43, 48, 49]. Overall, it has been suggested that older adults have a general interest in their health and fall risk [49], and self-control, independence and perceived need for safety are important motivations to use the technologies [48]. Additionally, cost and privacy have also been reported as important elements that ensure continued use of technologies among older adults [48]. It is also important to consider that older adults do not want equipment to identify them as ‘fallers’ or in need of help [48]. Consequently, proper ‘age-friendly’ branding and user-centered design need to be considered in future research. Regarding the clinician acceptance of sensing technology use, minimal preparation time, simple interface and real time result display have been reported as key factors for continued use of technologies [50]. The disconnect between clinical functionality and user experience evaluation remains a significant gap and warrants attention.

## Conclusions

To date, a wide range of sensor technologies have been utilized in fall risk assessment in older adults. Overall, these devices have the potential to provide an accurate, inexpensive, and easy-to-administer, objective fall risk assessment. However, the variation in measured parameters, assessment tools, sensor sites, movement tasks, and modelling techniques, precludes a firm conclusion on their ability to predict future falls. There is potential that these assessments can be undertaken regularly both in clinical and in non-clinical settings. To generate clinically meaningful and easy to interpret information, proper sensor-based predictors need to be identified. Additionally, a gap between clinical functionality and user experience remains.

## Abbreviations

5STS: Five Time Sit-to-Stand test
BBS: Berg Balance Scale
COM: Center of Mass
COP: Center of Pressure
KNN: k-Nearest Neighbor classifier
LWL: Locally Weighted Learning
MLP: Multi-layer perceptron classifier
NB: Naïve Bayesian classifier
NEAT: Neuro Evolution of Augmenting Topologies
PPA: Physiological Profile Assessment
RBFN: Radial Basis Function Network classifier
ROC: Receiver Operating Characteristic
SPPB: Short Physical Performance Battery
SVM: Support Vector Machine
Tinetti: Tinetti Performance Oriented Mobility Assessment
TUG: Timed Up and Go
